# CR TKA UHMWPE Wear Tested after Artificial Aging of the Vitamin E Treated Gliding Component by Simulating Daily Patient Activities

**DOI:** 10.1155/2014/567374

**Published:** 2014-11-20

**Authors:** Jens Schwiesau, Bernhard Fritz, Ines Kutzner, Georg Bergmann, Thomas M. Grupp

**Affiliations:** ^1^Aesculap AG, Research & Development, Am Aesculap Platz, 78532 Tuttlingen, Germany; ^2^Julius Wolff Institut, Charité-Universitätsmedizin Berlin, Augustenburger Platz 1, 13353 Berlin, Germany; ^3^Orthopedic Department, Ludwig-Maximilians-University, Campus Großhadern, Marchioninistraße 15, 81377 Munich, Germany

## Abstract

The wear behaviour of total knee arthroplasty (TKA) is dominated by two wear mechanisms: the abrasive wear and the delamination of the gliding components, where the second is strongly linked to aging processes and stress concentration in the material. The addition of vitamin E to the bulk material is a potential way to reduce the aging processes. This study evaluates the wear behaviour and delamination susceptibility of the gliding components of a vitamin E blended, ultra-high molecular weight polyethylene (UHMWPE) cruciate retaining (CR) total knee arthroplasty. Daily activities such as level walking, ascending and descending stairs, bending of the knee, and sitting and rising from a chair were simulated with a data set received from an instrumented knee prosthesis. After 5 million test cycles no structural failure of the gliding components was observed. The wear rate was with 5.62 ± 0.53 mg/million cycles falling within the limit of previous reports for established wear test methods.

## 1. Introduction

During the last decade the wear reduction of TKA was documented with wear tests simulating level walking [1]. Most of these tests have shown abrasive wear of the gliding components, also termed tibial inserts or articular surfaces. In contrast to clinical results a second wear phenomenon, delamination, occurred less frequently in simulations [2–4]. Delamination and cracks are fatigue failures of the gliding surfaces due to a combination of material aging and high stress concentration. Several test configurations have been proposed to generate delamination on TKA gliding components [5–11], but all of these attempts have the disadvantage of not having a homogeneous source of physiological loading data, requiring the application of several estimations. Nevertheless, new bearing materials for TKA should be analyzed for delamination susceptibility.

One possibility to reduce the risk of delamination for new bearing materials is to stabilize the mechanical properties by the prevention of aging. In regard to aging resistance, the addition of vitamin E has offered promising perspectives [12–14]. Aging of UHMWPE is related to a chemical reaction cascade between the macromolecules and oxygen. Irradiation processes from sterilisation or crosslinking generate free bonds (radicals) on the molecules. These bonds react with oxygen. One possible result of these reaction cascades can be a chain scission of the macromolecules leading to mechanical property degradation. Vitamin E can donate hydrogen to react with the free bonds and interrupt this reaction cascade.

Accelerated aging had no influence on the wear behaviour in a reciprocating unidirectional wear test [15] or a knee simulator study applying level walking [16] on UHMWPE stabilized with vitamin E. Stable mechanical properties after artificial aging [17] up to 4 weeks were reported [18, 19]. Most of the previous studies were conducted on highly crosslinked (>50 kGy) UHMWPE blended or diffusion treated with vitamin E. To the authors' knowledge vitamin E blended conventional UHWMPE irradiated with ~30 kGy for sterilisation has not been tested for reduced delamination risk after artificial aging.

## 2. Objective

The objective of our study was the application of highly demanding daily patient activities on a cruciate retaining knee design to evaluate the influence of artificial aging on the wear behavior of gliding components made from Vitamin E blended UHMWPE.

## 3. Materials and Methods

The wear test was performed on medium size Columbus CR TKA (Aesculap AG Tuttlingen, Germany) with the thinnest available gliding surface. Femoral components size F4 Left articulated against Vitamin E blended and artificially aged ultra high molecular weight polyethylene gliding components size T3. The gliding components were 10 mm high and fixed on a tibia tray. Femoral components and tibia trays made out of CoCr29Mo alloy were used. UHMWPE bulk was compression molded GUR 1020 resin blended with 0.1% vitamin E. For sterilization the gliding components were irradiated with 30 ± 2 kGy. Artificially aging was applied according to ASTM F2003-2 [17] afterwards for two week. An Oxidation Index of 0.1 was detected after this treatment. Prior to wear testing the gliding components were soaked in test lubricant at 37°C for 70 days to avoid influence from fluid absorption during the wear test. Hydration of nonsoaked specimens during the initial test interval can affect the wear result [20].

The simulation was performed on a load controlled 4 station knee wear simulator (EndoLab Thansau, Germany) capable of reproducing loads and movement of highly demanding daily activities. As reported previously the relative error (1.8% to 13.3%) for the loading components (flexion, axial load, internal-external torque, and anterior-posterior load) of the different activities results in a sufficient standard deviation of the resulting movements [8, 21]. In this setup the axial load, the flexion angle, the anterior-posterior load, and the internal-external torque are controlled; all other degrees of freedom are unrestrained. The neutral position is adjusted by self-alignment of the TKA during axial load in full extension (0° flexion). The load distribution was adapted from ISO 14243-1:2009(E) with 60% load medial and 40% load lateral for all activities. The simulation of the surrounding structures in an anterior cruciate ligament scarified knee was also adapted from ISO 14243-1:2009(E). The posterior tibia shift was restrained with 44 N/mm, the anterior tibia shift with 9.3 N/mm, and the internal-external rotation with 0.15 Nm/°. To avoid luxation there was no gap between the specimens and the restraining system.

**Figure 1:**
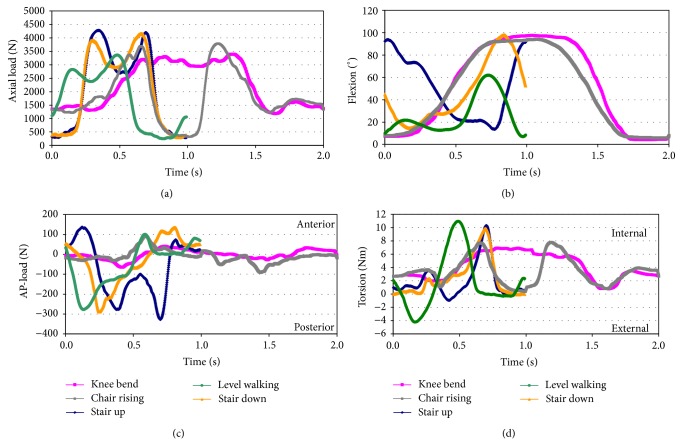
Axial load, flexion angle, anterior-posterior load, and internal-external torque applied during the simulation of knee bend, stair ascent and descent, sitting and rising from a chair, and level walking. The coordinate system from ISO 14243-1:2009(E) is applied [23]. First peak of the axial load for level walking, stair ascent and descent (coincident with contralateral foot lift). Increased axial load for knee bend corresponds to descent. Minimum axial load for sitting and rising from a chair indicates resting on the chair after sit-down. The timeframe was adapted from previously published data [59].

The applied profiles (Figure 1) were derived from flexion angle and load data of 8 subjects with implanted devices reported previously by Bergmann et al. [22]. These profiles are normalized to a patient weight of 100 kg as reported in the same study (“High100” loads) and converted to the coordinate system described in ISO 14243-1:2009(E) [23]. For walking, stair ascent and stair descent the cycle time was 1 s (1 Hz) [24] and, for the remaining high flexion activities, the cycle time was set to 2 s (0.5 Hz) [25]. The load profiles were applied in a loop consisting of 5 frames, 4000 cycles of stair descent, 4000 cycles of stair ascent, 200 cycles of deep squatting, 1000 cycles of level walking, and finally 800 cycles of sitting and rising from a chair. At the end of each frame the end of the load profile was directly applied to the start of the load profile in the next frame. The loop was repeated 500 times during the test. The enhanced application of high flexion activities compared to the outcome of studies of patient activities [24, 26] enabled a simulation of approximately 30 years in an average patient. The described procedure was previously applied in a wear test with highly demanding activities [8]. The load profiles and the movement of the TKA were recorded at a sampling rate of 500 Hz every 5000 cycles. Based on these records the patterns of load and motion are evaluated. In addition to the entire load profiles the maximum and minimum values for anterior-posterior displacement and internal-external rotation were recorded every 500 cycles. These data were used to calculate the average range of motion.

The tests were run in new born calf serum diluted with deionized water to a protein content of 20 g/L at 37°C [23]. The lubricant was stabilized with ethylene diamine tetraacetic acid (EDTA) to avoid precipitation of calcium phosphate and Amphotericin B to avoid microbiological contamination. The lubricant was changed every 500,000 test cycles. An axial loaded, soaked control specimen was used to detect lubricant absorption during the test [23]. After 0.5, 1.0, 2.0, 3.0, 4.0, and 5.0 million test cycles the wear of the specimens was detected gravimetrically with an analytical balance (CPA225D, Sartorius Göttingen, Germany) with an accuracy of 0.01 mg. The wear rate was calculated according to ISO 14243-2:2009(E) [27] respecting air buoyancy. At the same intervals the bearing surfaces were inspected optically. To evaluate the geometrical changes during the test the specimens were scanned before and after the test with a 3D measuring machine with a resolution of less than 3.5 *μ*m (UMM850, Zeiss Oberkochen, Germany). At each scan a minimum of 7500 points on an equidistant grid covering the bearing areas of the gliding components were recorded. The scans were superimposed and the geometrical changes were calculated (Holos NT 2.4.12, Zeiss Oberkochen, Germany). The results are displayed in pseudocolors in a plane transversal view.

## 4. Results

**Figure 2:**
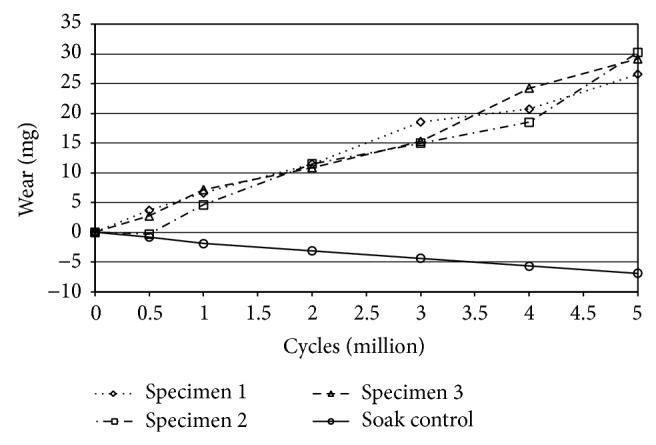
Gravimetric wear of the gliding surfaces during 5 million cycles of highly demanding activities.

An average wear rate of 5.62 ± 0.53 (standard deviation) mg/million cycles was detected for the vitamin E treated and artificially aged gliding components. The total weight loss after simulating 5 million cycles of high demanding activities was 26.60, 30.26, and 29.17 mg for the three individual specimens. The weight increase of the soak control during this time was 6.88 mg. The diagram of the weight measurement (Figure 2) shows a stable slope from the beginning throughout the duration of the test.

**Figure 3:**
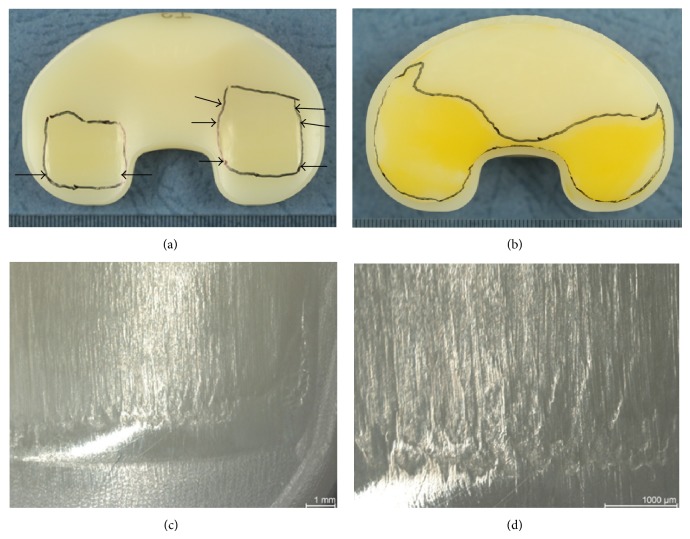
Proximal (a) and distal (b) surface of specimen 3 after 5 million cycles of highly demanding activities. (a) Worn areas on the proximal surface are framed; arrows indicate the region where striated patterns occurred. (b) The framed areas on the distal surface displayed a colour change and the machining marks disappeared. (c) Worn area on the dorsal lateral bearing with a polished transition between the machining marks and the striated patterns; region is indicated by the arrows in picture (a). (d) Magnification from (c) with striated patterns; scaling is 1 mm in all pictures.

Burnishing was the dominant wear pattern observed on the gliding surfaces. Striated pattern was observed mainly in medial-lateral orientation. Until the end of the test no crack formation, pitting, or delamination was observed. On the posterior distal surface the machining marks disappeared and the color changed (Figure 3).

**Figure 4:**
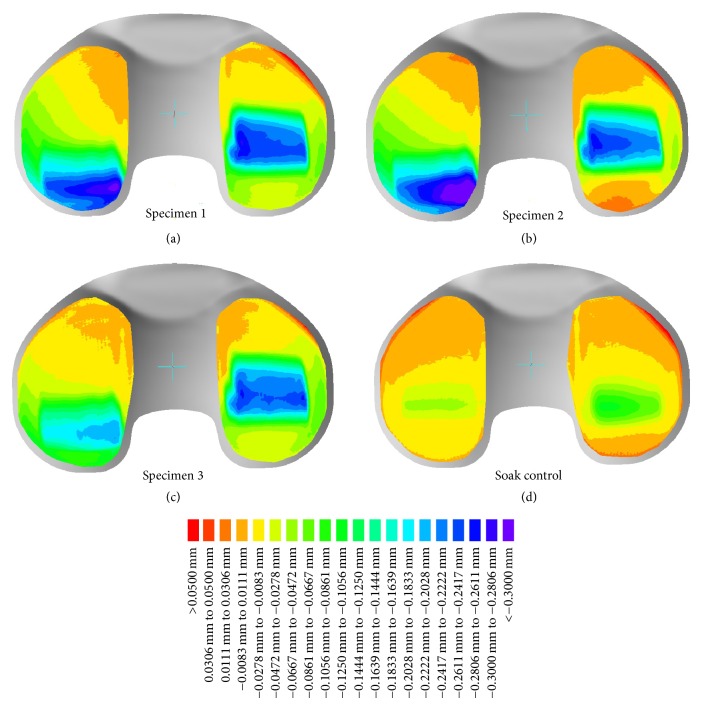
Geometrical changes after 5 million cycles of highly demanding activities, scale: red > 0.05 mm and purple < 0.30 mm.

Geometrical changes (Figure 4) show that the penetration on the worn specimens 1–3 was mainly dorsal on the lateral bearing, and central on the medial bearing. Penetration depth up to 0.3 mm occurred. The soak control revealed plastic deformation up to 0.1 mm due to compressive loads only.

**Figure 5:**
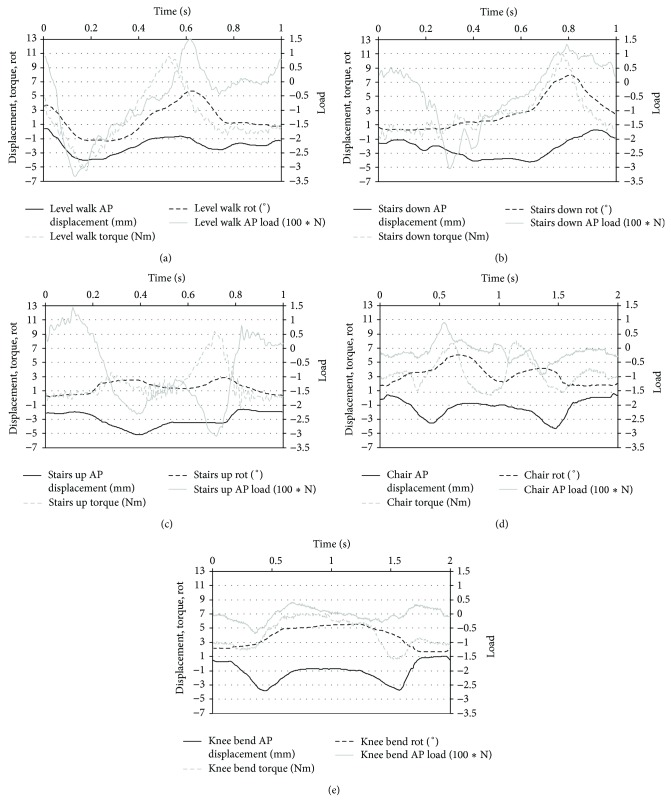
Patterns of anterior-posterior displacement with applied load and internal-external rotational with applied torque for the different activities at 0.5 million cycles. The coordinate system from ISO 14243-1:2009(E) is applied [23]. The time frame corresponds to Figure 1.

**Figure 6:**
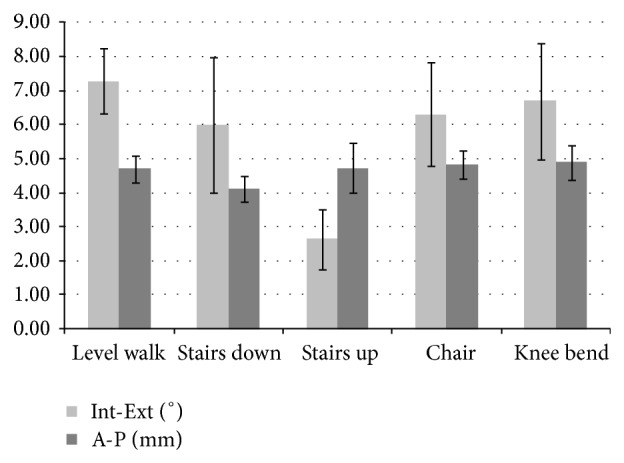
Range of internal-external rotation and anterior-posterior displacement for the simulated activities (average ± standard deviation), calculated by subtracting the minimum values from the maximum values recorded every 500 cycles.

Examples of the kinematic pattern extracted from the data recorded during the testing period up to 0.5 million cycles for anterior-posterior displacement and internal-external rotation are shown in Figure 5. For the activities with a frequency of 0.5 Hz the nominal and actual values are in good analogy. The activities with a frequency of 1.0 Hz have a higher deviation between nominal and actual values with partly high frequent noise. Nevertheless, this noise is not transformed in movement due to the inert mass of the simulator and the actual values are still in an acceptable range as reported previously [8]. There is a good correspondence between the direction of the applied load or torque and the direction of movement for level walking, stair descent, stair ascent, and sitting rising from a chair. The anterior-posterior displacement for all activities during the test ranged from 4 mm to 5 mm. The internal-external rotation ranged from 6° to 7° except for the simulation of stair ascent, which has less than half the rotation of all other activities. This is confirmed by the evaluation of the minimum and maximum values for anterior-posterior displacement and internal-external rotation for each activity during the testing period (Figure 6).

## 5. Discussion

Our objective was to evaluate the wear behavior of vitamin E treated TKR gliding components after artificial aging. This evaluation was based on a simulation of different patient activities with loading profiles recorded with instrumented TKA [22]. The crucial advantage of such data is the consistent source compared to previous studies with a similar objective [9, 21, 28].

**Figure 7:**
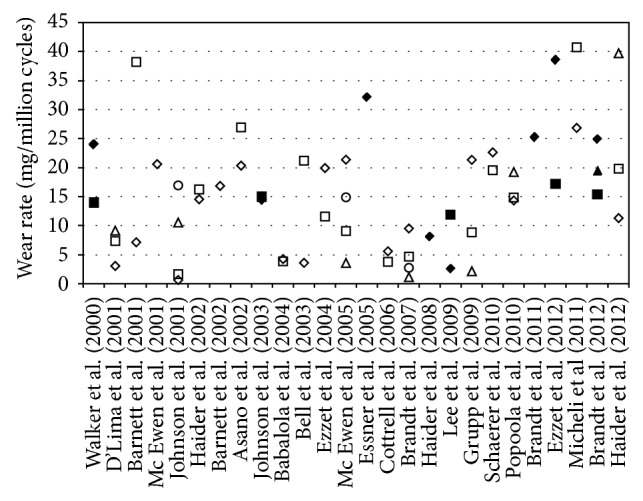
Overview of wear rates reported in the literature for clinically established CR knee implants. All results were obtained on conventional UHMWPE. Different symbols in a row indicate individual results in the same study. Dark symbols indicate different implant design; framed symbols indicate test modification (including aging) in the study.

In this study the gliding components had an average wear rate of 5.62 ± 0.53 mg/million cycles equivalent to 6.01 ± 0.50 mm^3^/million cycles assuming a material density of 0.935 [29] and a cumulative wear after 5 million cycles of 28.68 ± 1.88 mg (30.67 ± 2.00 mm^3^). This wear rate falls within the range of reports for clinically well-established CR TKA (Figure 7) [1, 10, 11, 16, 18, 30–50]. Compared to a previous test on conventional gliding surface material of the same design with the simulation of level walking only [1] the wear rate increased by a factor of 2.6.

Previous studies intended to evaluate the wear behavior of vitamin E treated UHMWPE simulated level walking only. Nevertheless, the wear rates reported in these studies are in a range comparable to the results of our test which includes more high demand daily activities.

Teramura et al. [51] reported with 27.4 mm^3^ a cumulated wear after 5 million simulated gait cycles for nonradiated GUR 1050 with 0.3 wt% vitamin E. In this test the displacement controlled load profile from ISO 14243-3:2004(E) [52] was adapted. A later study, Haider et al., tested different designs of crosslinked GUR 1020 after a diffusion treatment with vitamin E. Wear rates of 2.70 mg/million cycles, 5.98 mg/million cycles, and 3.06 mg/million cycles are reported for a large size of a PS and CR design and a small size of the same CR design [18]. In this study ISO 14243-1:2009(E) [23] was applied. Artificial aging of the gliding components according to ASTM F2003 [17] was conducted before wear testing a crosslinked UHMWPE containing vitamin E by Vaidya et al. [53]. They reported a wear rate of 1.9 ± 1.9 mg/million cycles. The wear behavior of 100 kGy crosslinked material soak treated in vitamin E was evaluated with and without artificial aging by Micheli et al. [16]. In this study a wear rate of 2.4 ± 0.5 mg/million cycles was reported for the unaged material and 2.5 ± 0.8 mg/million cycles for the artificially aged material. Similar to our results none of the vitamin E specimens from the previous studies have shown structural failure. The ability to generate structural fatigue (like cracks or delamination) with the current test protocol was demonstrated in a pilot study. There, a conventional UHMWPE showed delamination within the first million test cycles after it was initially aged for 3 weeks with the conditions described in ASTM F2003 [17]. This confirms previous wear test results for conventional Gamma sterilized UHMWPE after accelerated ageing [7]. Nevertheless, a recent report about wear tests with a simplified test setup indicates that shelf aging can have a severe influence on the delamination behavior compared to the accelerated aging according to the current standard [54]. The observed wear patterns on the proximal bearings are in good agreement with previous reports [55, 56]. The reason for the change in the colour on the distal surface of the gliding component can only be hypothesized at the moment. A possible explanation is given by Costa et al. and Serro et al. [57, 58]. They describe how substances from the lubricant can be absorbed on the surface and diffuse into the bulk material.

The geometrical changes show central penetration on the medial side and posterior penetration on the lateral side. This indicates that the main penetration occurred during internal rotation of the tibia. Furthermore, the penetration areas on specimen 3 and the soak control are more pronounced on the medial side reflecting the medially shifted load distribution during the test. Due to axial loading the soak control has a deformation of ~0.07 mm on the lateral side and ~0.1 mm on the medial side. This means that up to 30% of the penetration depth can be related to plastic deformation.

The internal rotation of the tibia during loading is confirmed by the kinematic analysis. Internal rotation occurred for all activities. Stair ascent rotation was limited by the form fit between femur and gliding surface in extension. The anterior-posterior displacement is aligned in the same direction as the applied anterior-posterior load. The range of motion for level walking is increased compared to a previous report [1] due to reduced stiffness of the restraining system for internal-external rotation but the anterior-posterior displacement is comparable for the two studies (4.8 ± 0.8 mm [1] versus 4.7 ± 0.4 mm).

## 6. Conclusions

Even with the simulated application of daily activities which exceeded the loading limit and period of currently standardized testing criteria, as well as further chemical treatment by artificial aging, the tested material has a wear rate within the limits of currently established materials and shows no indication of structural failure.
